# Adaptive Output Feedback Control for Nonholonomic Chained Systems with Integral Input State Stability Inverse Dynamics

**DOI:** 10.3390/s23146351

**Published:** 2023-07-13

**Authors:** Yang Li, Linxing Xu, Xiuli Wang, Cunsong Wang

**Affiliations:** 1 Shanghai Key Laboratory of Power Station Automation Technology, Shanghai University, Shanghai 200444, China; yongerli@shu.edu.cn; 2 School of Mechatronic Engineering and Automation, Shanghai University, Shanghai 200444, China; 3 College of Information Engineering, Zhejiang University of Technology, Hangzhou 310023, China; sherrywang@zjut.edu.cn; 4 Institute of Intelligent Manufacturing, Nanjing Tech University, Nanjing 210006, China; wangcunsong@njtech.edu.cn

**Keywords:** nonholonomic chained system, iISS inverse dynamics, input-state scaling, backstepping, switching control, reduced-order observer

## Abstract

This paper investigates a class of nonholonomic chained systems with integral input-to-state stable (iISS) inverse dynamics subject to unknown virtual control directions and parameter uncertainty included in drift terms. First, the system is divided into two interconnected subsystems according to the system’s structure. Second, one controller is designed using a switch strategy for state finite escape. Then, another controller and adaptive law are designed by combining a reduced-order state observer and backstepping method after input-state scaling. Finally, simulation results validate the feasibility of the proposed control algorithm.

## 1. Introduction

In recent years, the control of nonholonomic systems has drawn considerable attention due to the enormous potential practical values. And nonholonomic systems have penetrated deeply into humans’ daily lives, such as in the case of planar space robots, the knife edge, a rigid spacecraft with two torque actuators, cars towing several trailers and multiple mobile manipulators [1,2,3]. Some classical nonlinear control methods have been developed rapidly in recent years, such as recursive integral backstepping control [4], adaptive output feedback control [5], sliding mode control [6], etc. Control of nonholonomic systems has been booming for several years in the nonlinear control field. However, the stabilization problem for nonholonomic systems, while the systems are subject to nonholonomic constraints, makes it difficult to utilize the classical control methods of nonlinear systems [7]. It is further proved by Brockett’s necessary condition that the equilibrium of a simple nonholonomic system could not be stabilized by any static continuous state feedback control even for a controllable open loop system [8]. Alternatively, time-varying controllers and discontinuous controllers have been developed for nonholonomic systems’ stability. For example, ref. [9] investigated the state feedback and output feedback to address the effects of nonholonomic constraints in event-triggered control. Ref. [10] developed a continuous state feedback control strategy for first-order nonholonomic systems.

It is well-recognized that a lot of nonholonomic systems could be transformed into their corresponding chained forms. Therefore, nonholonomic systems in a chained form as fundamental systems have been taken much more seriously [11]. Furthermore, the design of adaptive controllers for nonholonomic systems with unknown control directions is a considerable concerned problem [12]. The adaptive control problem becomes more difficult when the sign of virtual control is unknown. Robust control is a common strategy for the stability problem of nonholonomic systems with unknown control direction [13]. This method first designs a controller according to the assumed control direction, and then identifies the control direction online. If the actual control direction is the opposite of the assumed direction, then a continuous switching law is applied to modify the controller.

Unlike previous works, in this paper, a class of integral Input state stability (iISS) inverse dynamics is introduced to a canonical chained nonholonomic system with parameter uncertainties, which is shown as follows:

(1) $$\begin{array}{cc} \; & \dot{\eta }=q\left(\eta ,x_{0}\right),\\ \; & {\dot{x}}_{0}=d_{0}u_{0}+\phi_{0}^{T}\left(\eta ,x_{0}\right)\theta ,\\ \; & {\dot{x}}_{1}=d_{1}u_{0}x_{2}+\phi_{1}^{T}\left(u_{0},\eta ,x_{0},x_{1}\right)\theta ,\\ \; & {\dot{x}}_{i}=d_{i}u_{0}x_{i+1}+\phi_{i}^{T}\left(u_{0},\eta ,x_{0},x\right)\theta ,\\ & i=2,\cdots ,n-1\\ \; & {\dot{x}}_{n}=d_{n}u_{1}+\phi_{n}^{T}\left(u_{0},\eta ,x_{0},x\right)\theta ,\\ \; & y=(x_{0},x_{1}), \end{array}$$

where $u_{0}$ and $u_{1}$ represent the control inputs; ${\left[x_{0},x^{T}\right]}^{T}={\left[x_{0},x_{1},\cdots ,x_{n}\right]}^{T}\in {ℜ}^{n+1}$ and $\eta \in {ℜ}^{q}$ are system states; η is referred to inverse dynamics and is available to control design; *y* denotes the measurable output; $q\left(\eta ,x_{0}\right)\in {ℜ}^{q}$ is locally Lipschitz and holds q(0,0)=0. $\phi_{0}\left(\eta ,x_{0}\right)\in {ℜ}^{l}$, $\phi_{1}\left(u_{0},\eta ,x_{0},x_{1}\right)\in {ℜ}^{l}$ and $\phi_{i}\left(u_{0},\eta ,x_{0},x\right)\in {ℜ}^{l}\;\left(2\leq i\leq n\right)$ are smooth vector functions and satisfy $\phi_{0}\left(0,0\right)=0$, $\phi_{i}\left(u_{0},0,0,0\right)=0\;\left(1\leq i\leq n\right)$; $\theta \in {ℜ}^{l}$ shows a vector of unknown constant parameters; $d_{0},\cdots ,d_{n}$ indicate virtual control directions while $d_{1},\cdots ,d_{n}$ are unknown but bounded.

ilSS was first defined in [14], and is strictly weaker than ISS. The properties of iISS are further ventilated in [15]. ISS is a nonlinear extension on the finite gain of the maximum norm and $L_{2}$, replacing finite linear gains that require too much general nonlinear control with nonlinear gains. When excited by a uniformly bounded energy signal, a system exhibits a low energy response, which is a highly demanding qualitative characteristic. In this case, a weaker condition iISS is proposed naturally and applied to nonlinear systems with good performance [16,17]. As iISS inverse dynamics are first introduced into nonholonomic systems with unknown control direction and strong drift, two interconnected subsystems are divided according to the system’s structure. Then, one controller is designed by a switch strategy for state finite escape. Another controller is designed by combining a reduced-order state observer and backstepping method after input-state scaling. Generally, the control problem of interest is to design adaptive output feedback controllers $u_{0}=u_{0}\left(\eta ,x_{0},\hat{\Theta }\right),u_{1}=u_{1}\left(\eta ,x_{0},x,\hat{\Theta },\mu \right),\mu =v\left(\eta ,x_{0},x,\hat{\Theta },\mu \right)$ to make the components of closed-loop system bounded and η(t), $x_{0}\left(t\right)$, x(t) converge to its equilibrium when t→∞ on the condition that the inverse dynamics η is iISS.

The rest of this paper is organized as follows. Section 2 presents the essential preliminary results. An output feedback controller is provided in Section 3 with the aid of Section 3.1 controller design for $u_{0}$ and Section 3.2 controller design for $u_{1}$. Section 4 addresses the switch control. Section 5 illustrates the effectiveness of the proposed algorithm.

## 2. Preliminary Results

Consider the following nonlinear control system

(2) $$\begin{array}{ccc} \dot{x} & = & f\left(x,u\right),\;x\in {ℜ}^{n},\;u\in {ℜ}^{m} \end{array}$$

where *u* denotes the control input, *x* represents the state and *f* satisfying f(0,0)=0 is a locally Lipschitz function. Next, let us recall several important definitions and propositions.

**Definition** **1** ([18])**.** *A continuous function $\Psi_{1}:\left[0,a\right)\to \left[0,\infty \right)$ is said to be a K-function if it is strictly increasing and $\Psi_{1}\left(0\right)=0$. The K-function $\Psi_{1}$ is called a $K_{\infty }$-function if a=∞ and $\Psi_{1}\left(r\right)\to \infty$ as r→∞.*

**Definition** **2** ([18])**.** *A continuous function $\Psi_{2}:\left[0,a\right)\times \left[0,\infty \right)\to \left[0,\infty \right)$ is said to be a KL-function if, for every fixed s, the mapping $\Psi_{2}\left(r,s\right)$ is a K-function with respect to r and, for every fixed r, the mapping $\Psi_{2}\left(r,s\right)$ is decreasing with respect to s and $\Psi_{2}\left(r,s\right)\to 0$ as s→∞.*

**Definition** **3** ([19])**.** *System (2) is called integral input-to-state stable (iISS) with respect to u, if for any initial value $x\left(0\right)\in {ℜ}^{n}$ there always exist $K_{\infty }$-functions α, $\gamma_{\alpha }$ and a KL-function $\beta_{\alpha }$ such that*

(3) $$\begin{array}{ccc} \alpha (|x(t)|) & \leq & \beta_{\alpha }\left(|x\left(0\right)|,t\right)+\int_{0}^{t}\gamma_{\alpha }\left(|u\left(\tau \right)|\right)d\tau . \end{array}$$

The definition of iISS could be equivalently illustrated by the Lyapunov function in the following proposition.

**Proposition** **1** ([19])**.** *System (2) is iISS if and only if there is a positive definite and proper function V(x), referred to an iISS-Lyapunov function, such that*

(4) $$\begin{array}{c} \underline{\alpha }\left(|x|\right)\leq V\left(x\right)\leq \bar{\alpha }\left(|x|\right),\\ \frac{\partial V}{\partial x}\left(x\right)f\left(x,u\right)\leq -\beta \left(|x|\right)+\gamma \left(|u|\right), \end{array}$$

*where $\underline{\alpha }$, $\bar{\alpha }$ and γ are $K_{\infty }$-functions, β represents a positive definite continuous function.*

Before the following proportion, there is a notation for simplicity that $\sigma_{1}\left(s\right)=O\left(\sigma_{2}\left(s\right)\right)$ denotes $\sigma_{1}\left(s\right)\leq c\sigma_{2}\left(s\right)$ for some constant c>0 and any *s* in a small neighborhood of the origin.

**Proposition** **2** ([20])**.** *Consider the system (2) with an iISS-Lyapunov function V(x) satisfying (4). If any smooth function π with the property $\pi^{2}\left(s\right)=O\left(\beta \left(s\right)\right)$ is taken. And the additional condition $\underset{s\to \infty }{lim sup}\frac{\pi^{2}\left(s\right)}{\beta (s)}<\infty$ holds when β is bounded. Then there always exists a positive definite function σ and a $K_{\infty }$-function ω such that*

(5) $$\begin{array}{ccc} \int_{0}^{t}\pi^{2}\left(|x\left(\tau \right)|\right)d\tau & \leq & \sigma \left(|x\left(0\right)|\right)+\int_{0}^{t}\omega \left(|u\left(\tau \right)|\right)d\tau , \end{array}$$

(6) $$\begin{array}{ccc} \int_{0}^{t}|u\left(\tau \right)|\pi (|x\left(\tau \right)|)d\tau & \leq & \sigma \left(|x\left(0\right)|\right)+\int_{0}^{t}\left(\omega (|u\left(\tau \right)|\right)+\frac{1}{4}{\left|u\left(\tau \right)\right|}^{2})d\tau . \end{array}$$

*Furthermore, if the iISS-gain γ satisfies $\gamma \left(s\right)=O\left(s^{2}\right)$, so is ω.*

Now we start to investigate the nonholonomic system (1). Before the output controller design, there are some commonly used hypotheses.

**Assumption** **A1.** *There is a constraint of $d_{1},\cdots ,d_{n}$ that $d_{1}\cdots d_{n}=1$. Furthermore, for nonlinear vector function $\phi_{0}\left(\eta ,x_{0}\right)$, there exists a nonnegative smooth function ${\bar{\phi }}_{0}\left(\eta ,x_{0}\right)$ such that $|\phi_{0}\left(\eta ,x_{0}\right)\left|\leq \right|x_{0}|{\bar{\phi }}_{0}\left(\eta ,x_{0}\right)$.*

**Assumption** **A2.** *There exist continuously differential matrix functions ${\bar{\phi }}_{i}^{T}\left(u_{0},\eta ,x_{0},\bar{x}\right)\in {ℜ}^{1\times m_{i}}$ (i=2,⋯,n) and continuous matrix functions $\varphi_{i}\left(d_{1},\cdots ,d_{n-1}\right)\in {ℜ}^{m_{i}\times l}\;\left(i=2,\cdots ,n\right)$, where $\bar{x}=\left[{\bar{x}}_{1},\cdots ,{\bar{x}}_{n}\right]$ and*

(7) $$\begin{array}{c} \begin{array}{cc} & {\bar{x}}_{1}=x_{1},\\ & {\bar{x}}_{i}=d_{1}\cdots d_{i-1}x_{i}.\;i=2,\cdots ,n \end{array} \end{array}$$

*such that $\phi_{i}^{T}(u_{0},\eta ,x_{0},$ $\bar{x},d_{1},\cdots ,d_{n-1})\;\left(i=2,\cdots ,n\right)$ satisfy $\phi_{i}^{T}(u_{0},\eta ,x_{0},\bar{x},d_{1},\cdots ,$ $d_{n-1})={\bar{\phi }}_{i}^{T}\left(u_{0},\eta ,x_{0},\bar{x}\right)$ $\varphi_{i}\left(d_{1},\cdots ,d_{n-1}\right)$.*

**Remark** **1.** *Transformation (7) takes a major effect in tackling uncertainties in system (1). However, the unknown parameters are brought into $\phi_{i}^{T}\left(u_{0},\eta ,x_{0},\bar{x}\right)\;\left(i=2,\cdots ,n\right)$ by (7). There is no doubt that it becomes more complicated to estimate the uncertainties. So Assumption 2 plays an active part in separating uncertainties out from $\phi_{i}^{T}\left(u_{0},\eta ,x_{0},\bar{x}\right)\;\left(i=2,\cdots ,n\right)$.*

According to transformation (7) and Assumption 2, system (1) can be converted into:

(8) $$\begin{array}{c} \begin{array}{cc} & \dot{\eta }=q\left(\eta ,x_{0}\right),\\ & {\dot{x}}_{0}=d_{0}u_{0}+\phi_{0}^{T}\left(\eta ,x_{0}\right)\theta ,\\ & {\dot{\bar{x}}}_{1}=u_{0}{\bar{x}}_{2}+{\bar{\phi }}_{1}^{T}\left(u_{0},\eta ,x_{0},{\bar{x}}_{1}\right)\theta ,\\ & {\dot{\bar{x}}}_{i}=u_{0}{\bar{x}}_{i+1}+{\bar{\phi }}_{i}^{T}\left(u_{0},\eta ,x_{0},\bar{x}\right)\Theta_{i},\\ & i=2,\cdots ,n-1\\ & {\dot{\bar{x}}}_{n}=u_{1}+{\bar{\phi }}_{n}^{T}\left(u_{0},\eta ,x_{0},\bar{x}\right)\Theta_{n}, \end{array} \end{array}$$

where ${\bar{\phi }}_{1}^{T}\left(u_{0},\eta ,x_{0},{\bar{x}}_{1}\right)=\phi_{1}^{T}\left(u_{0},\eta ,x_{0},x_{1}\right)$, $\Theta_{i}=d_{1}\cdots d_{i-1}\varphi_{i}\left(d_{0},\cdots ,d_{n-1}\right)\theta \in {ℜ}^{m_{i}\times 1}\left(2\leq i\leq n\right)$.

## 3. Output Feedback Controller

For system (8), an adaptive output feedback controller will be designed via input-state scaling transformation and backstepping technique. In detail, this system will be divided into two subsystems around its inherent structure. First, $u_{0}$ will be designed to stabilize the $\left(\eta ,x_{0}\right)$-subsystem under the condition that η is iISS. And then, the stability of $\bar{x}-$ subsystem is guaranteed by controller $u_{1}\left(t\right)$ to be framed later. For clarity, the discussion will be separated into two categories: $x_{0}\left(0\right)=0$ and $x_{0}\left(0\right)\neq 0$. This section is concerned with the condition of $x_{0}\left(0\right)\neq 0$.

### 3.1. Controller Design for $u_{0}$

For subsystem

(9) $$\begin{array}{ccc} \dot{\eta } & = & q(\eta ,x_{0}), \end{array}$$

(10) $$\begin{array}{ccc} {\dot{x}}_{0} & = & d_{0}u_{0}+\phi_{0}^{T}\left(\eta ,x_{0}\right)\theta , \end{array}$$

the following sufficient conditions are required:

(A1) System (9) is iISS, i.e., there is an iISS-Lyapunov function V(η), such that

(11) $$\begin{array}{cc} \; & \underline{\alpha }\left(|\eta |\right)\leq V\left(\eta \right)\leq \bar{\alpha }\left(|\eta |\right),\\ \; & \frac{\partial V(\eta )}{\partial \eta }q\left(\eta ,x_{0}\right)\leq -\beta \left(|\eta |\right)+\gamma (|x_{0}\left|\right), \end{array}$$

where $\underline{\alpha }$, $\bar{\alpha }$ and γ are $K_{\infty }$-functions, β represents a positive definite continuous function.

(A2) There is two unknown positive constants $p_{01}$, $p_{02}$, and two known positive semidefinite smooth functions $\phi_{01}$, $\phi_{02}$ such that

(12) $$\begin{array}{ccc} |\phi_{0}^{T}\left(\eta ,x_{0}\right){\hat{\theta }}_{0}| & \leq & p_{01}\phi_{01}\left(\right|x_{0}\left|,\right|{\hat{\theta }}_{0}\left|\right)+p_{02}\phi_{02}\left(|\eta |\right), \end{array}$$

where ${\hat{\theta }}_{0}$ denotes the estimate of unknown parameter θ in $x_{0}$-subsystem; ${\tilde{\theta }}_{0}$ is the corresponding estimate error and satisfies ${\tilde{\theta }}_{0}=\theta -{\hat{\theta }}_{0}$. Then controller $u_{0}$ and adaptive law ${\dot{\hat{\theta }}}_{0}$ are given in the succeeding theorem.

**Theorem** **1.** *Suppose that the conditions A1) and A2) hold with the properties $\phi_{02}^{2}\left(s\right)=O\left(\beta \left(s\right)\right)$ and $\gamma \left(s\right)=O\left(s^{2}\right)$. When β is bounded, there is $\underset{s\to \infty }{lim sup}\frac{\phi_{02}^{2}\left(s\right)}{\beta (s)}<\infty$. If the control law and adaptive law are chosen as:*

(13) $$\begin{array}{ccc} u_{0} & = & -cd_{0}\kappa \varphi_{0}\left(x_{0},{\hat{\theta }}_{0}\right)x_{0},\;\dot{\kappa }=\Gamma \varphi_{0}\left(x_{0},{\hat{\theta }}_{0}\right)x_{0}^{2},\;\kappa \left(0\right)>0, \end{array}$$

(14) $$\begin{array}{ccc} {\dot{\hat{\theta }}}_{0} & = & x_{0}\phi_{0}\left(\eta ,x_{0}\right)≜\beta_{0}, \end{array}$$

*then by appropriately selecting positive smooth function $\varphi_{0}$ and positive parameters c, Γ, we obtain:*

*(i) the solution of the closed-loop system (9), (), (13) and () are well-defined and bounded over [0,∞).*

*(ii)For any initial states,*

(15) $$\begin{array}{ccc} \lim_{t\to \infty }\left(\right|x_{0}\left(t\right)|+|\eta \left(t\right)|+|u_{0}\left(t\right)\left|\right) & = & 0. \end{array}$$

**Proof.**  (i) For subsystem (10), a candidate Lyapunov function is chosen as $V_{0}\left(x_{0}\right)=\frac{1}{2}x_{0}^{2}+\frac{1}{2}{\tilde{\theta }}_{0}^{T}{\tilde{\theta }}_{0}$. The time derivative of $V(x_{0})$ along trajectories (10), (13) and (14) is granted by

(16) $$\begin{array}{ccc} {\dot{V}}_{0}\left(x_{0}\right) & = & x_{0}\left(-cd_{0}^{2}\kappa \varphi_{0}\left(x_{0},{\hat{\theta }}_{0}\right)x_{0}+\phi_{0}^{T}\left(\eta ,x_{0}\right)\left({\tilde{\theta }}_{0}+{\hat{\theta }}_{0}\right)\right)+{\tilde{\theta }}_{0}^{T}{\dot{\tilde{\theta }}}_{0}\\ & = & -cd_{0}^{2}\kappa \varphi_{0}\left(x_{0},{\hat{\theta }}_{0}\right)x_{0}^{2}+x_{0}\phi_{0}^{T}\left(\eta ,x_{0}\right){\hat{\theta }}_{0}\\ & \leq & -cd_{0}^{2}\kappa \varphi_{0}\left(x_{0},{\hat{\theta }}_{0}\right)x_{0}^{2}+p_{01}|x_{0}|\phi_{01}\left(\right|x_{0}\left|,\right|{\hat{\theta }}_{0}\left|\right)+p_{02}|x_{0}|\phi_{02}\left(|\eta |\right). \end{array}$$

According to Proposition 2, we can achieve

(17) $$\begin{array}{ccc} \int_{0}^{t}|x_{0}\left(\tau \right)|\phi_{02}\left(|\eta \left(\tau \right)|\right)d\tau & \leq & \sigma_{0}\left(|\eta \left(0\right)|\right)+\int_{0}^{t}\omega_{0}\left(\right|x_{0}\left(\tau \right)\left|\right)d\tau , \end{array}$$

where $\sigma_{0}$ is a positive definite function, $\omega_{0}\in K_{\infty }$ and satisfies $\omega_{0}\left(s\right)=O\left(s^{2}\right)$. Select a positive smooth function $\varphi_{0}$ such that

(18) $$\begin{array}{ccc} x_{0}^{2}\varphi_{0}\left(x_{0},{\hat{\theta }}_{0}\right) & \geq & \max\{|x_{0}|\phi_{01}\left(\right|x_{0}\left|,\right|{\hat{\theta }}_{0}|),\omega_{0}\left(\right|x_{0}\left|),\gamma (\right|x_{0}|)\}. \end{array}$$

The existence of function $\varphi_{0}$ is guaranteed by the fact that $\phi_{01}$ is a positive semidefinite smooth function, $\omega_{0}$ and γ are $K_{\infty }$- functions. Then with (13), (18) and (16) can be rewritten as

(19) $$\begin{array}{ccc} {\dot{V}}_{0} & \leq & -\frac{c}{\Gamma }d_{0}^{2}\kappa \dot{\kappa }+\frac{p_{01}}{\Gamma }\dot{\kappa }+p_{02}|x_{0}|\phi_{02}\left(|\eta |\right). \end{array}$$

Integrating both side of (19), it yields

(20) $$\begin{array}{ccc} V_{0}\left(t\right)-V_{0}\left(0\right) & \leq & -\frac{c}{2\Gamma }d_{0}^{2}\kappa^{2}\left(t\right)+\frac{p_{01}+p_{02}}{\Gamma }\kappa \left(t\right)+b, \end{array}$$

where $b=\frac{c}{2\Gamma }d_{0}^{2}\kappa^{2}\left(0\right)-\frac{p_{01}+p_{02}}{\Gamma }\kappa \left(0\right)+p_{02}\sigma_{0}\left(|\eta \left(0\right)|\right)$.

It is assumed that the solutions of the closed-loop system are defined on a right-maximal interval [0,T) with 0<T≤∞. κ(t) will be proved to be bounded on [0,T) by contradiction. Suppose that κ(t) is unbounded. Since $\dot{\kappa }\left(t\right)\geq 0$ that κ(t) is an increasing function and κ(t)→∞ when t→∞. Upon division of both side of (20), we obtain

(21) $$\begin{array}{ccc} \frac{-V_{0}\left(0\right)-b}{\kappa (t)}-\frac{p_{01}+p_{02}}{\Gamma } & \leq & -\frac{c}{2\Gamma }d_{0}^{2}\kappa^{2}\left(t\right). \end{array}$$

Obviously, the left hand of (21) tends to a finite number while the right tends to −∞. It leads to a contradiction. Therefore, It reveals that κ(t) is bounded on [0,T).

According to (13) and (18) and the boundness of κ(t), it easily draw a conclusion that $\int_{0}^{t}\gamma (|x_{0}\left(\tau \right)\left|\right)d\tau$ is bounded on interval [0,T). Moreover, using (11), it follows that η defined on interval [0,T) is bounded. Based on (20) and the boundness of κ, we also obtain that $x_{0}$ and ${\hat{\theta }}_{0}$ are bounded. Finally, T=∞ can be similarly proved by the proof of Proposition 6 in [21]. The proof of (i) is completed.

(ii) The boundness of $x_{0}\left(t\right)$ and ${\dot{x}}_{0}\left(t\right)$ imply that $\gamma (|x_{0}\left(t\right)|)$ is uniformly continuous on [0,∞). Furthermore, the property $\int_{0}^{\infty }\gamma (|x_{0}\left(\tau \right)\left|\right)d\tau$ and Barbalat Lemma yield $\lim_{t\to \infty }\gamma (|x_{0}\left(t\right)\left|\right)=0$. This, in turn, indicates $\lim_{t\to \infty }\left|x_{0}\left(t\right)\right|=0$, $\lim_{t\to \infty }\left|u_{0}\left(t\right)\right|=0$. Then, by applying $\int_{0}^{\infty }\gamma (|x_{0}\left(\tau \right)\left|\right)d\tau$ and Proposition 6 in [21], it follows that η→0 as t→∞. Finally, the whole proof of Theorem 1 is completed. □

**Remark** **2.** *If a nonnegative function is chosen as $W_{0}\left(x_{0}\right)=\frac{1}{2}x_{0}^{2}$, its time derivative could be arranged into the form of ${\dot{W}}_{0}\left(x_{0}\right)\geq W_{0}\left(x_{0}\right)\lambda \left(t\right)$ coupled with (13) and Assumption 1, where $\lambda \left(t\right)=-2(cd_{0}^{2}\kappa \varphi_{0}\left(x_{0},{\hat{\theta }}_{0}\right)+{\bar{\phi }}_{0}\left(\eta ,x_{0}\right)|\theta |)$. Then, it is easy to note that $W_{0}\left(x_{0}\left(t\right)\right)\geq W_{0}\left(x_{0}\left(0\right)\right)$ $\exp(\int_{0}^{t}\lambda \left(\tau \right)d\tau )>0$ by Comparison Lemma. In this case, we arrive at a straightforward conclusion that $u_{0}\left(t\right)\neq 0$ when $x_{0}\left(0\right)\neq 0$. As a result, it is of interest to interpret that the input-state technique to be introduced below is available.*

From the above analysis, we are actually aware that the state of $x_{0}$-subsystem could be regulated to zero. But $\bar{x}$-subsystem is uncontrollable. In order to avoid this phenomenon, a discontinuous input-state scaling transformation is proposed:

(22) $$\begin{array}{ccc} z_{i} & = & \frac{{\bar{x}}_{i}}{u_{0}^{n-i}},\;i=1,\cdots ,n-1,\;z_{n}={\bar{x}}_{n}. \end{array}$$

Under the *z* coordination, $\bar{x}$-subsystem is rearranged into

(23) $$\begin{array}{cc} \; & {\dot{z}}_{1}=z_{2}-\left(n-1\right)\frac{f_{1}}{u_{0}}z_{1}-\left(n-1\right)\frac{f_{2}}{u_{0}}z_{1}\theta +\psi_{1}^{T}\left(u_{0},\eta ,x_{0},z_{1}\right)\theta ,\\ \; & {\dot{z}}_{i}=z_{i+1}-\left(n-i\right)\frac{f_{1}}{u_{0}}z_{i}-\left(n-i\right)\frac{f_{2}}{u_{0}}z_{i}\theta +\psi_{i}^{T}\left(u_{0},\eta ,x_{0},z_{1},\cdots ,z_{n}\right)\Theta_{i},\\ \; & i=2,\cdots ,n-1\\ \; & {\dot{z}}_{n}=u_{1}+\psi_{n}^{T}\left(u_{0},\eta ,x_{0},z_{1},\cdots ,z_{n}\right)\Theta_{n}, \end{array}$$

where $f_{1}=\frac{\partial u_{0}}{\partial x_{0}}d_{0}u_{0}+\frac{\partial u_{0}}{\partial {\hat{\theta }}_{0}}\beta_{0}+\frac{\partial u_{0}}{\partial \kappa }\Gamma \varphi_{0}\left(x_{0},{\hat{\theta }}_{0}\right)x_{0}^{2}$, $f_{2}=\frac{\partial u_{0}}{\partial x_{0}}\phi_{0}^{T}\left(\eta ,x_{0}\right)$, $\psi_{1}^{T}\left(u_{0},\eta ,x_{0},z_{1}\right)=\frac{{\bar{\phi }}_{1}^{T}\left(u_{0},\eta ,x_{0},{\bar{x}}_{1}\right)}{u_{0}^{n-1}}$, $\psi_{i}^{T}\left(u_{0},\eta ,x_{0},z_{1},\cdots ,z_{n}\right)=\frac{{\bar{\phi }}_{i}^{T}\left(u_{0},\eta ,x_{0},\bar{x}\right)}{u_{0}^{n-i}},\;i=2,\cdots ,n$.

### 3.2. Controller Design for $u_{1}$

Since states $x_{0},$ $x_{1}$ are measurable and $u_{0}$ is definitely given by Theorem 1 that state $z_{1}$ which is obtained by transformations $z_{1}=\frac{x_{1}}{u_{0}^{n-1}}$ and ${\bar{x}}_{1}=x_{1}$ is measurable. Therefore, a reduced-order state observer could be proposed for system (23). For notation convenience, we covert the $\left(z_{2},\cdots ,z_{n}\right)$-subsystem into its compact form:

(24) $$\begin{array}{ccc} \dot{z} & = & \left(A-\frac{f_{1}}{u_{0}}L\right)z+bu_{1}-\frac{f_{2}}{u_{0}}\theta Lz+\psi^{T}\Theta , \end{array}$$

where $z=\left(\begin{array}{c} z_{2}\\ \vdots \\ z_{n} \end{array}\right)$, $\Theta =\left(\begin{array}{c} \Theta_{2}\\ \vdots \\ \Theta_{n} \end{array}\right)$, $A=\left(\begin{array}{cccccc} 0 & 1 & 0 & \cdots & 0 & 0\\ 0 & 0 & 1 & \cdots & 0 & 0\\ \vdots & \vdots & \vdots & & \vdots & \vdots \\ 0 & 0 & 0 & \cdots & 0 & 1\\ 0 & 0 & 0 & \cdots & 0 & 0 \end{array}\right)$, $b=\left(\begin{array}{c} 0\\ \vdots \\ 0\\ 1 \end{array}\right)$, $\psi^{T}=\left(\begin{array}{cccc} \psi_{2}^{T} & 0 & \cdots & 0\\ 0 & \psi_{3}^{T} & \cdots & 0\\ \vdots & \vdots & & \vdots \\ 0 & 0 & \cdots & \psi_{n}^{T} \end{array}\right)$, $L=\left(\begin{array}{ccccc} n-2 & 0 & \cdots & 0 & 0\\ 0 & n-3 & \cdots & 0 & 0\\ \vdots & \vdots & & \vdots & \vdots \\ 0 & 0 & \cdots & 1 & 0\\ 0 & 0 & \cdots & 0 & 0 \end{array}\right)$. In addition, ${\hat{z}}_{2},\cdots ,{\hat{z}}_{n}$ denote the estimate of states $z_{2},\cdots ,z_{n}$. *e* is the estimate error and satisfies the equation $e=col\left(z_{2},\cdots ,z_{n}\right)-col\left({\hat{z}}_{2},\cdots ,{\hat{z}}_{n}\right)$. $\hat{\theta }$ and $\hat{\Theta }$ represent the estimates of θ and Θ. $\tilde{\theta }$ and $\tilde{\Theta }$ are the responding estimate errors, and satisfy $\tilde{\theta }=\theta -\hat{\theta }$, $\tilde{\Theta }=\Theta -\hat{\Theta }$, respectively. ${\hat{\psi }}^{T}\left(u_{0},\eta ,x_{0},z_{1},{\hat{z}}_{2},\cdots ,{\hat{z}}_{n}\right)$ is the estimate of $\psi^{T}\left(u_{0},\eta ,x_{0},z_{1},z_{2},\cdots ,z_{n}\right)$. It is certainly useful to make several assumptions in the following part.

**Assumption** **A3.** *There are continuous bounded matrix functions $K_{1}\left(t\right)\in {ℜ}^{\left(n-1)\times (n-1\right)}$, $K_{2}\left(t\right)\in {ℜ}^{\left(n-1)\times (n-1\right)}$,$H_{1}\left(t\right)\in {ℜ}^{(n-1)\times l}$, $H_{2}\left(t\right)\in {ℜ}^{\left(n-1\right)\times \sum_{i=2}^{n}m_{i}}$, such that $\frac{f_{2}}{u_{0}}\theta Lz-\frac{f_{2}}{u_{0}}\hat{\theta }L\hat{z}=K_{1}\left(t\right)e+H_{1}\left(t\right)\tilde{\theta }$, $\psi^{T}\Theta -{\hat{\psi }}^{T}\hat{\Theta }=K_{2}\left(t\right)e+H_{2}\left(t\right)\tilde{\Theta }$, and $H_{1}\left(t\right)\to 0$, $H_{2}\left(t\right)\to 0$, when t→∞.*

**Assumption** **A4.** *Matrix $A-\frac{f_{1}}{u_{0}}L-K_{1}\left(t\right)+K_{2}\left(t\right)$ is negative definite, which makes the following n−1 dimension reduced-order state observer feasible.*

We now establish the n−1 dimension reduced-order state observer

(25) $$\begin{array}{ccc} \dot{\hat{z}} & = & \left(A-\frac{f_{1}}{u_{0}}L\right)\hat{z}+bu_{1}-\frac{f_{2}}{u_{0}}\hat{\theta }L\hat{z}+\hat{\psi^{T}}\hat{\Theta }+\zeta_{1}^{T}\dot{\hat{\theta }}-\zeta_{2}^{T}\dot{\hat{\Theta }}, \end{array}$$

where $\zeta_{1}^{T}={\left[\zeta_{12},\cdots ,\zeta_{1n}\right]}^{T}\in {ℜ}^{(n-1)\times l}$ and $\zeta_{2}^{T}={\left[\zeta_{22},\cdots ,\zeta_{2n}\right]}^{T}\in {ℜ}^{\left(n-1\right)\times \sum_{i=2}^{n}m_{i}}$ are, respectively, generated by

(26) $$\begin{array}{c} {\dot{\zeta_{1}}}^{T}=\left(A-\frac{f_{1}}{u_{0}}L-K_{1}\left(t\right)+K_{2}\left(t\right)\right){\zeta_{1}}^{T}-H_{1}\left(t\right),\\ {\dot{\zeta_{2}}}^{T}=\left(A-\frac{f_{1}}{u_{0}}L-K_{1}\left(t\right)+K_{2}\left(t\right)\right){\zeta_{2}}^{T}-H_{2}\left(t\right). \end{array}$$

Under the condition of (24), (25) and Assumption 3, we obtain the following differential equation of observer error vector *e* after a simple manipulator

(27) $$\begin{array}{ccc} \dot{e} & = & \left(A-\frac{f_{1}}{u_{0}}L-K_{1}\left(t\right)+K_{2}\left(t\right)\right)e-H_{1}\left(t\right)\tilde{\theta }+H_{2}\left(t\right)\tilde{\Theta }-\zeta_{1}^{T}\dot{\hat{\theta }}+\zeta_{2}^{T}\dot{\hat{\Theta }}. \end{array}$$

If we perform $\tilde{e}=e-\zeta_{1}^{T}\tilde{\theta }+\zeta_{2}^{T}\tilde{\Theta }$ and substitute (26) into it, a new differential equation is achieved

(28) $$\begin{array}{ccc} \dot{\tilde{e}} & = & \bar{A}\tilde{e}, \end{array}$$

where $\bar{A}=A-\frac{f_{1}}{u_{0}}L-K_{1}\left(t\right)+K_{2}\left(t\right)$. Furthermore, according to Assumption 4, a positive parameter δ is properly chosen such that the following linear matrix inequality(LMI) holds

(29) $$\begin{array}{ccc} P\bar{A}+{\bar{A}}^{T}P+\frac{n}{4}\delta^{-1}I & \leq & -Q, \end{array}$$

where P,Q are positive definite matrix; *I* denotes identity matrix. Now we can rewrite the feedback control system into

(30) $$\begin{array}{cc} \; & \dot{\tilde{e}}=\bar{A}\tilde{e},\\ \; & {\dot{z}}_{1}={\hat{z}}_{2}+{\tilde{e}}_{2}+\zeta_{12}^{T}\tilde{\theta }-\zeta_{22}^{T}\tilde{\Theta }-\left(n-1\right)\frac{f_{1}}{u_{0}}z_{1}-\left(n-1\right)\frac{f_{2}}{u_{0}}z_{1}\theta +\psi_{1}^{T}\theta ,\\ \; & {\dot{\hat{z}}}_{i}={\hat{z}}_{i+1}-\left(n-i\right)\frac{f_{1}}{u_{0}}\hat{z_{i}}-\left(n-i\right)\frac{f_{2}}{u_{0}}\hat{z_{i}}\hat{\theta }+\Phi_{i}\hat{\Theta }+\zeta_{1i}^{T}\dot{\hat{\theta }}-\zeta_{2i}^{T}\dot{\hat{\Theta }},\\ \; & i=2,\cdots ,n-1\\ \; & {\dot{\hat{z}}}_{n}=u_{1}+\Phi_{n}\hat{\Theta }-\zeta_{2n}^{T}\dot{\hat{\Theta }}, \end{array}$$

where $\Phi_{i}=\left[0,\cdots ,0,{\hat{\psi }}_{i}^{T},0,\cdots ,0\right]$, i=2,⋯,n.

**Remark** **3.** *There are always solutions for LMI (29) by appropriately choosing δ. For example, if matrix Q>0 is arbitrarily given, we can select a suitable δ so that a positive definite matrix P exists and satisfies inequality (29). On the other hand, we can also solve the inequality (29) by LMI tool box. According to Schur Lemma [22], (29) can be rewritten into its LMI form*

(31) $$\begin{array}{cc} \left(\begin{array}{cc} P\bar{A}+{\bar{A}}^{T}P+Q & I\\ I & -\frac{4}{n}\delta I \end{array}\right)\leq & 0. \end{array}$$

*Then we can arrive at P,Q by LMI toolbox in Matlab.*

Now, we are in a position to sketch the controller of the system (30) based on the backstepping technique.

**Step 1.** Consider $\left(\tilde{e},\xi_{1}\right)$-subsystem and define new state variables $\xi_{1}=z_{1}$, $\xi_{2}={\hat{z}}_{2}-\alpha_{1}$, where $\alpha_{1}\left(\eta ,x_{0},{\hat{\theta }}_{0},\kappa ,\xi_{1},\hat{\theta }\right)$ is applied as a virtual control input. Our candidate Lyapunov function is introduced as

(32) $$\begin{array}{ccc} V_{1} & = & {\tilde{e}}^{T}P\tilde{e}+\frac{1}{2}\xi_{1}^{2}+\frac{1}{2}{\tilde{\theta }}^{T}\tilde{\theta }+\frac{1}{2}{\tilde{\Theta }}^{T}\tilde{\Theta } \end{array}$$

Then its derivative ${\dot{V}}_{1}$ with respect to *t* is

(33) $$\begin{array}{ccc} {\dot{V}}_{1} & = & {\tilde{e}}^{T}\left(P\bar{A}+{\bar{A}}^{T}P\right)\tilde{e}+\xi_{1}\xi_{2}+\xi_{1}\left(\alpha_{1}-\left(n-1\right)\frac{f_{1}}{u_{0}}\xi_{1}-\left(n-1\right)\frac{f_{2}}{u_{0}}\xi_{1}\hat{\theta }+\psi_{1}^{T}\hat{\theta }\right)+\xi_{1}{\tilde{e}}_{2}\\ & & +{\tilde{\theta }}^{T}\left(\dot{\tilde{\theta }}+\xi_{1}\zeta_{12}-\left(n-1\right)\xi_{1}^{2}\frac{f_{2}^{T}}{u_{0}}+\xi_{1}\psi_{1}\right)+{\tilde{\Theta }}^{T}\left(\dot{\tilde{\Theta }}-\xi_{1}\zeta_{22}\right). \end{array}$$

Using the property of perfect square yields $\xi_{1}{\tilde{e}}_{2}\leq \frac{1}{4}\delta^{-1}{\tilde{e}}^{T}\tilde{e}+\delta \xi_{1}^{2}$. If virtual control law α and tuning functions $\beta_{1}$, $\tau_{1}$ of adaptive control law $\dot{\hat{\theta }}$, $\dot{\hat{\Theta }}$ are picked as

(34) $$\begin{array}{ccc} \alpha_{1} & = & -p_{1}\xi_{1}-\delta \xi_{1}+\left(n-1\right)\frac{f_{1}}{u_{0}}\xi_{1}+\left(n-1\right)\frac{f_{2}}{u_{0}}\xi_{1}\hat{\theta }-\psi_{1}^{T}\hat{\theta }, \end{array}$$

(35) $$\begin{array}{ccc} \beta_{1} & = & \xi_{1}\zeta_{12}-\left(n-1\right)\xi_{1}^{2}\frac{f_{2}^{T}}{u_{0}}+\xi_{1}\psi_{1},\;\tau_{1}=-\xi_{1}\zeta_{22}, \end{array}$$

where $p_{1}>0$, then ${\dot{V}}_{1}$ is rendered into

(36) $$\begin{array}{ccc} {\dot{V}}_{1} & \leq & {\tilde{e}}^{T}\left(P\bar{A}+{\bar{A}}^{T}P+\frac{1}{4}\delta^{-1}I\right)\tilde{e}-p_{1}\xi_{1}^{2}+\xi_{1}\xi_{2}+{\tilde{\theta }}^{T}\left(\dot{\tilde{\theta }}+\beta_{1}\right)+{\tilde{\Theta }}^{T}\left(\dot{\tilde{\Theta }}+\tau_{1}\right). \end{array}$$

It is obvious that $\alpha_{1}\left(\eta ,x_{0},{\hat{\theta }}_{0},\kappa ,\xi_{1},\hat{\theta }\right)$ is a smooth function and satisfies $\alpha_{1}\left(0,0,{\hat{\theta }}_{0},\kappa ,0,\hat{\theta }\right)=0$.

**Step i (2≤i≤n−1).** For system $\left(\tilde{e},\xi_{1},\cdots ,\xi_{i}\right)$, redefine state variable $\xi_{i+1}={\hat{z}}_{i+1}-\alpha_{i}$, where $\alpha_{i}\left(\eta ,x_{0},{\hat{\theta }}_{0},\kappa ,\xi_{1},\cdots ,\xi_{i},\hat{\theta },\hat{\Theta }\right)$ is the virtual control input to be designed in step i. Before step i, we have already received the smooth virtual control input $\alpha_{i-1}$ and

(37) $${\dot{V}}_{i-1}\leq {\tilde{e}}^{T}\left(P\bar{A}+{\bar{A}}^{T}P+\frac{i-1}{4}\delta^{-1}I\right)\tilde{e}-\sum_{j=1}^{i-1}p_{j}\xi_{j}^{2}+\xi_{i-1}\xi_{i}+{\tilde{\theta }}^{T}\left(\dot{\tilde{\theta }}+\beta_{i-1}\right)+{\tilde{\Theta }}^{T}\left(\dot{\tilde{\Theta }}+\tau_{i-1}\right).$$

Now, we augment $V_{i-1}$ with a quadratic term of state $\xi_{i}$ to acquire the Lyapunov function $V_{i}=V_{i-1}+\frac{1}{2}\xi_{i}^{2}$. Its derivative is

(38) $$\begin{array}{cc} {\dot{V}}_{i} & \leq {\tilde{e}}^{T}\left(P\bar{A}+{\bar{A}}^{T}P+\frac{i-1}{4}\delta^{-1}I\right)\tilde{e}-\sum_{j=1}^{i-1}p_{j}\xi_{j}^{2}+\xi_{i}\xi_{i+1}+\xi_{i}(\alpha_{i}+\xi_{i-1}-\left(n-i\right)\frac{f_{1}}{u_{0}}\left(\xi_{i}+\alpha_{i-1}\right)\\ \; & \;-\left(n-i\right)\frac{f_{2}}{u_{0}}\left(\xi_{i}\;+\;\alpha_{i-1}\right)\hat{\theta }\;+\;\Phi_{i}\hat{\Theta }\;+\;\zeta_{1i}^{T}\dot{\hat{\theta }}\;-\;\zeta_{2i}^{T}\dot{\hat{\Theta }}\;-\;\frac{\partial \alpha_{i-1}}{\partial \eta }q\left(\eta ,x_{0}\right)\;-\;\frac{\partial \alpha_{i-1}}{\partial x_{0}}\left(d_{0}u_{0}\;+\;\phi_{0}^{T}\hat{\theta }\right)\;-\;\frac{\partial \alpha_{i-1}}{\partial {\hat{\theta }}_{0}}\beta_{0}\\ \; & -\frac{\partial \alpha_{i-1}}{\partial \kappa }\Gamma \varphi_{0}x_{0}^{2}-\frac{\partial \alpha_{i-1}}{\partial \xi_{1}}\left(\xi_{2}+\alpha_{1}-\left(n-1\right)\frac{f_{1}}{u_{0}}\xi_{1}-\left(n-1\right)\frac{f_{2}}{u_{0}}\xi_{1}\hat{\theta }+\psi_{1}^{T}\hat{\theta }\right)-\sum_{j=2}^{i-1}\frac{\partial \alpha_{i-1}}{\partial {\hat{z}}_{j}}({\hat{z}}_{j+1}\\ \; & \;-\;\left(n\;-\;j\right)\frac{f_{1}}{u_{0}}{\hat{z}}_{j}\;-\;\left(n\;-\;j\right)\frac{f_{2}}{u_{0}}{\hat{z}}_{j}\hat{\theta }\;+\;\Phi_{j}\hat{\Theta }\;+\;\zeta_{1j}^{T}\dot{\hat{\theta }}\;-\;\zeta_{2j}^{T}\dot{\hat{\Theta }})\;-\;\frac{\partial \alpha_{i-1}}{\partial \hat{\theta }}\dot{\hat{\theta }}\;-\;\frac{\partial \alpha_{i-1}}{\partial \hat{\Theta }}\dot{\hat{\Theta }})\;-\;\xi_{i}\frac{\partial \alpha_{i-1}}{\partial \xi_{1}}{\tilde{e}}_{2}\;+\;{\tilde{\theta }}^{T}(\dot{\tilde{\theta }}\\ \; & +\beta_{i-1}-\xi_{i}\frac{\partial \alpha_{i-1}}{\partial x_{0}}\phi_{0}-\xi_{i}\frac{\partial \alpha_{i-1}}{\partial \xi_{1}}\left(\zeta_{12}-\left(n-i\right)\xi_{1}\frac{f_{2}^{T}}{u_{0}}+\psi_{1}\right))+{\tilde{\Theta }}^{T}\left(\dot{\tilde{\Theta }}+\tau_{i-1}+\xi_{i}\frac{\partial \alpha_{i-1}}{\partial \xi_{1}}\zeta_{22}\right). \end{array}$$

With the help of perfect square property, it holds

(39) $$\begin{array}{ccc} -\xi_{i}\frac{\partial \alpha_{i-1}}{\partial \xi_{1}}{\tilde{e}}_{2} & \leq & \frac{1}{4}\delta^{-1}{\tilde{e}}^{T}\tilde{e}+\delta {\left(\frac{\partial \alpha_{i-1}}{\partial \xi_{1}}\right)}^{2}\xi_{i}^{2}, \end{array}$$

We will attempt to employ the following control law $\alpha_{i}$ and tuning functions to cope with (38)

(40) $$\begin{array}{cc} \alpha_{i} & =\;-\;p_{i}\xi_{i}\;-\;\delta {\left(\frac{\partial \alpha_{i-1}}{\partial \xi_{1}}\right)}^{2}\xi_{i}\;-\;\xi_{i-1}\;+\;\left(n-i\right)\frac{f_{1}}{u_{0}}\left(\xi_{i}\;+\;\alpha_{i-1}\right)\;+\;\left(n-i\right)\frac{f_{2}}{u_{0}}\left(\xi_{i}\;+\;\alpha_{i-1}\right)\hat{\theta }\;-\;\Phi_{i}\hat{\Theta }\;-\;\zeta_{1i}^{T}\dot{\hat{\theta }}\\ \; & +\zeta_{2i}^{T}\dot{\hat{\Theta }}+\frac{\partial \alpha_{i-1}}{\partial \eta }q\left(\eta ,x_{0}\right)+\frac{\partial \alpha_{i-1}}{\partial x_{0}}\left(d_{0}u_{0}+\phi_{0}^{T}\hat{\theta }\right)+\frac{\partial \alpha_{i-1}}{\partial {\hat{\theta }}_{0}}\beta_{0}+\frac{\partial \alpha_{i-1}}{\partial \kappa }\Gamma \varphi_{0}x_{0}^{2}+\frac{\partial \alpha_{i-1}}{\partial \xi_{1}}(\xi_{2}+\alpha_{1}\\ \; & -\left(n-1\right)\frac{f_{1}}{u_{0}}\xi_{1}-\left(n-1\right)\frac{f_{2}}{u_{0}}\xi_{1}\hat{\theta }+\psi_{1}^{T}\hat{\theta })+\sum_{j=2}^{i-1}\frac{\partial \alpha_{i-1}}{\partial {\hat{z}}_{j}}({\hat{z}}_{j+1}-\left(n-j\right)\frac{f_{1}}{u_{0}}{\hat{z}}_{j}-\left(n-j\right)\frac{f_{2}}{u_{0}}{\hat{z}}_{j}\hat{\theta }\\ \; & +\Phi_{j}\hat{\Theta }+\zeta_{1j}^{T}\dot{\hat{\theta }}-\zeta_{2j}^{T}\dot{\hat{\Theta }})+\frac{\partial \alpha_{i-1}}{\partial \hat{\theta }}\dot{\hat{\theta }}+\frac{\partial \alpha_{i-1}}{\partial \hat{\Theta }}\dot{\hat{\Theta }}, \end{array}$$

(41) $$\begin{array}{cc} \beta_{i} & =\beta_{i-1}-\xi_{i}\frac{\partial \alpha_{i-1}}{\partial x_{0}}\phi_{0}-\xi_{i}\frac{\partial \alpha_{i-1}}{\partial \xi_{1}}\left(\zeta_{12}-\left(n-i\right)\xi_{1}\frac{f_{2}^{T}}{u_{0}}+\psi_{1}\right),\;\tau_{i}=\tau_{i-1}+\xi_{i}\frac{\partial \alpha_{i-1}}{\partial \xi_{1}}\zeta_{22}, \end{array}$$

where $p_{i}>0$. It is interesting to note that $\alpha_{i}\left(\eta ,x_{0},{\hat{\theta }}_{0},\xi_{1},\cdots ,\xi_{i},\hat{\theta },\hat{\Theta }\right)$ is a smooth function and satisfies $\alpha_{i}\left(0,0,{\hat{\theta }}_{0},0,\cdots ,0,\hat{\theta },\hat{\Theta }\right)=0$. Eventually, in view of (39)–(41), it is easy to show that

(42) $$\begin{array}{ccc} {\dot{V}}_{i} & \leq & {\tilde{e}}^{T}\left(P\bar{A}+{\bar{A}}^{T}P+\frac{i}{4}\delta^{-1}I\right)\tilde{e}-\sum_{j=1}^{i}p_{j}\xi_{j}^{2}+\xi_{i}\xi_{i+1}+{\tilde{\theta }}^{T}\left(\dot{\tilde{\theta }}+\beta_{i}\right)+{\tilde{\Theta }}^{T}\left(\dot{\tilde{\Theta }}+\tau_{i}\right). \end{array}$$

**Step n.** Investigate the system $\left(\tilde{e},\xi_{1},\cdots ,\xi_{n}\right)$, where state variable $\xi_{n}={\hat{z}}_{n}-\alpha_{n-1}$, the smooth virtual control input $\alpha_{n-1}\left(\eta ,x_{0},{\hat{\theta }}_{0},\xi_{1},\cdots ,\xi_{n-1},\hat{\theta },\hat{\Theta }\right)$ is already designed in step n−1. The basic idea of step n is to exploit the real control law $u_{1}$ and adaptive laws $\dot{\hat{\theta }}$, $\dot{\hat{\Theta }}$ to guarantee that the derivative of the suitable Lyapunov function is nonpositive. Now let us introduce the augmented candidate Lyapunov function $V_{n}=V_{n-1}+\frac{1}{2}\xi_{n}^{2}$. With repeated application of ${\dot{V}}_{n-1}$, its time derivative along trajectory is

(43) $$\begin{array}{cc} {\dot{V}}_{n} & \leq {\tilde{e}}^{T}\left(P\bar{A}\;+\;{\bar{A}}^{T}P\;+\;\frac{n-1}{4}\delta^{-1}I\right)\tilde{e}\;-\;\sum_{j=1}^{n-1}p_{j}\xi_{j}^{2}\;+\;\xi_{n}(u_{1}\;+\;\xi_{n-1}\;+\;\Phi_{n}\hat{\Theta }\;-\;\zeta_{2n}^{T}\dot{\hat{\Theta }}\;-\;\frac{\partial \alpha_{n-1}}{\partial \eta }q\left(\eta ,x_{0}\right)\\ \; & -\frac{\partial \alpha_{n-1}}{\partial x_{0}}\left(d_{0}u_{0}+\phi_{0}^{T}\hat{\theta }\right)-\frac{\partial \alpha_{n-1}}{\partial {\hat{\theta }}_{0}}\beta_{0}-\frac{\partial \alpha_{n-1}}{\partial \kappa }\Gamma \varphi_{0}x_{0}^{2}-\frac{\partial \alpha_{n-1}}{\partial \xi_{1}}(\xi_{2}+\alpha_{1}-\left(n-1\right)\frac{f_{1}}{u_{0}}\xi_{1}-(n\\ \; & \;-\;1)\frac{f_{2}}{u_{0}}\xi_{1}\hat{\theta }\;+\;\psi_{1}^{T}\hat{\theta })\;-\;\sum_{j=2}^{n-1}\frac{\partial \alpha_{n-1}}{\partial {\hat{z}}_{j}}\left({\hat{z}}_{j+1}\;-\;\left(n-j\right)\frac{f_{1}}{u_{0}}{\hat{z}}_{j}-\left(n-j\right)\frac{f_{2}}{u_{0}}{\hat{z}}_{j}\hat{\theta }\;+\;\Phi_{j}\hat{\Theta }\;+\;\zeta_{1j}^{T}\dot{\hat{\theta }}-\zeta_{2j}^{T}\dot{\hat{\Theta }}\right)\\ \; & -\frac{\partial \alpha_{n-1}}{\partial \hat{\theta }}\dot{\hat{\theta }}-\frac{\partial \alpha_{n-1}}{\partial \hat{\Theta }}\dot{\hat{\Theta }})-\xi_{n}\frac{\partial \alpha_{n-1}}{\partial \xi_{1}}{\tilde{e}}_{2}+{\tilde{\theta }}^{T}(\dot{\tilde{\theta }}+\beta_{n-1}-\xi_{n}\frac{\partial \alpha_{n-1}}{\partial x_{0}}\phi_{0}-\xi_{n}\frac{\partial \alpha_{n-1}}{\partial \xi_{1}}(\zeta_{12}\\ \; & -\left(n-1\right)\xi_{1}\frac{f_{2}^{T}}{u_{0}}+\psi_{1}))+{\tilde{\Theta }}^{T}\left(\dot{\tilde{\Theta }}+\tau_{n-1}+\xi_{n}\frac{\partial \alpha_{n-1}}{\partial \xi_{1}}\zeta_{22}\right). \end{array}$$

Similarly, by the choice of control law

(44) $$\begin{array}{cc} u_{1} & =\;-\;p_{n}\xi_{n}\;-\;\delta {\left(\frac{\partial \alpha_{n-1}}{\partial \xi_{1}}\right)}^{2}\xi_{n}-\xi_{n-1}\;-\;\Phi_{n}\hat{\Theta }\;+\;\zeta_{2n}^{T}\dot{\hat{\Theta }}+\frac{\partial \alpha_{n-1}}{\partial \eta }q\left(\eta ,x_{0}\right)+\frac{\partial \alpha_{n-1}}{\partial x_{0}}\left(d_{0}u_{0}+\phi_{0}^{T}\hat{\theta }\right)\\ \; & +\frac{\partial \alpha_{n-1}}{\partial {\hat{\theta }}_{0}}\beta_{0}+\frac{\partial \alpha_{n-1}}{\partial \kappa }\Gamma \varphi_{0}x_{0}^{2}+\frac{\partial \alpha_{n-1}}{\partial \xi_{1}}\left(\xi_{2}+\alpha_{1}-\left(n-1\right)\frac{f_{1}}{u_{0}}\xi_{1}-\left(n-1\right)\frac{f_{2}}{u_{0}}\xi_{1}\hat{\theta }+\psi_{1}^{T}\hat{\theta }\right)\\ \; & \;+\;\sum_{j=2}^{n-1}\frac{\partial \alpha_{n-1}}{\partial {\hat{z}}_{j}}\left({\hat{z}}_{j+1}\;-\;\left(n\;-\;j\right)\frac{f_{1}}{u_{0}}{\hat{z}}_{j}\;-\;\left(n\;-\;j\right)\frac{f_{2}}{u_{0}}{\hat{z}}_{j}\hat{\theta }\;+\;\Phi_{j}\hat{\Theta }\;+\;\zeta_{1j}^{T}\dot{\hat{\theta }}\;-\;\zeta_{2j}^{T}\dot{\hat{\Theta }}\right)+\frac{\partial \alpha_{n-1}}{\partial \hat{\theta }}\dot{\hat{\theta }}+\frac{\partial \alpha_{n-1}}{\partial \hat{\Theta }}\dot{\hat{\Theta }}, \end{array}$$

where $p_{n}>0$, and adaptive laws

(45) $$\begin{array}{ccc} \dot{\hat{\theta }} & = & \beta_{n-1}-\xi_{n}\frac{\partial \alpha_{n-1}}{\partial x_{0}}\phi_{0}-\xi_{n}\frac{\partial \alpha_{n-1}}{\partial \xi_{1}}\left(\zeta_{12}-\left(n-1\right)\xi_{1}\frac{f_{2}^{T}}{u_{0}}+\psi_{1}\right),\\ \;\dot{\hat{\Theta }} & = & \tau_{n-1}+\xi_{n}\frac{\partial \alpha_{n-1}}{\partial \xi_{1}}\zeta_{22}, \end{array}$$

the time derivative becomes ${\dot{V}}_{n}\leq -{\tilde{e}}^{T}Q\tilde{e}-\sum_{j=1}^{n}p_{j}\xi_{j}^{2}$ and is guaranteed to be nonpositive.

The analysis of the adaptive controller will be further extended into the following theorem.

**Theorem** **2.** *For x-system in (1), if the controller and adaptive controllers are established as (44) and (45) under the assumption of 3 and 4, then the x-system is globally regulated at the origin when $x_{0}\left(0\right)\neq 0$.*

**Proof.**  Under Theorem 1, we can conclude that $\lim_{t\to \infty }x_{0}=0$. Then for subsystem (30), it is easy to testify that $\tilde{e},z_{1},{\hat{z}}_{i}\;\left(i=2,\cdots ,n\right),\;\tilde{\theta },\tilde{\Theta }$ are bounded and $\lim_{t\to \infty }\left(\right|\tilde{e}\left|+\right|z_{1}|+\sum_{i=2}^{n}|{\hat{z}}_{i}\left|\right)=0$. In turn, the fact that $\tilde{e}=e-\zeta_{1}^{T}\tilde{\theta }+\zeta_{2}^{T}\tilde{\Theta }$, $e=col\left(z_{2},\cdots ,z_{n}\right)-col\left({\hat{z}}_{2},\cdots ,{\hat{z}}_{n}\right)$, Assumption 4 and (26) implies $\lim_{t\to \infty }\left(\right|\tilde{e}|+\sum_{i=1}^{n}|z_{i}\left|\right)=0$. Finally, from the transformations (7) and (22), we can prove that $\lim_{t\to \infty }\sum_{i=1}^{n}\left|x_{i}\right|=0$. So the *x*-system is globally regulated at the origin when $x_{0}\left(0\right)\neq 0$. □

## 4. Switching Controller

As we all know, $u_{0}$ is commonly applied as a constant controller in multiple works when $x_{0}\left(0\right)=0$. However, in this paper, due to the nonlinearity $\phi_{0}^{T}\left(\eta ,x_{0}\right)\theta$ in the first equation of (8), finite escape may occur in subsystem $x_{0}$. To avoid this phenomenon, we set

(46) $$\begin{array}{ccc} u_{0} & = & -cd_{0}\kappa \varphi_{0}\left(x_{0},{\hat{\theta }}_{0}\right)x_{0}+\eta_{0},\\ \; & if & -cd_{0}\kappa \varphi_{0}\left(x_{0},{\hat{\theta }}_{0}\right)x_{0}+\eta_{0}\geq u_{0}^{*}\\ u_{0} & = & -cd_{0}\kappa \varphi_{0}\left(x_{0},{\hat{\theta }}_{0}\right)x_{0},\;else\\ \dot{\kappa } & = & \Gamma \varphi_{0}\left(x_{0},{\hat{\theta }}_{0}\right)x_{0}^{2},\\ {\dot{\hat{\theta }}}_{0} & = & x_{0}\phi_{0}\left(\eta ,x_{0}\right), \end{array}$$

where $0<u_{0}^{*}<\eta_{0}$. The positive constant $\eta_{0}$ in $u_{0}$ drives the state $x_{0}\left(t\right)$ away from zero. Assuming that $t=t^{*}$ when $u_{0}=u_{0}^{*}$, it can be known that $x_{0}\left(t\right)$ is bounded for $0\leq t<t^{*}$. We also learn that $x_{0}\left(t\right)$ is bounded and $\lim_{t\to \infty }\left(\right|x_{0}\left(t\right)|+|\eta \left(t\right)|+|u_{0}\left(t\right)\left|\right)=0$ for $t\geq t^{*}$ according to the similar procedure of proof in theorem 1. Therefore, $x_{0}\left(t\right)$ will not blow up under the control (46) when $x_{0}\left(0\right)=0$. Next, it is easy to show that $x_{0}\left(t\right)$ does not cross zero by the fact of Remark 2. Meanwhile, the control input $u_{1}\left(t\right)$ is essentially designed as same as the case of $x_{0}\left(0\right)\neq 0$.

The above analysis is summarized into the following theorem.

**Theorem** **3.** For system (8), if the control inputs and adaptive controllers are organized as (46), $u_{1}=u_{1}^{*}\left(u_{0},\eta ,x_{0},z,\hat{\theta },\hat{\Theta }\right)$, $\dot{\hat{\theta }}=\beta_{n}^{*}$, $\dot{\hat{\Theta }}=\tau_{n}^{*}$ under assumptions listed above, then the reconstructed closed-loop system is globally regulated at the origin when $x_{0}\left(0\right)=0$.

## 5. Simulation Example

In this section, a three-dimensional nonholonomic system with iISS inverse dynamics is proposed to illustrate the effectiveness of the derived control laws.

(47) $$\begin{array}{ccc} \dot{\eta } & = & -\frac{\eta }{1+\eta^{2}}+x_{0}^{2},\\ {\dot{x}}_{0} & = & d_{0}u_{0}+\frac{1}{2}x_{0}+\frac{1}{2}x_{0}\frac{\eta }{1+\eta^{2}},\\ {\dot{x}}_{1} & = & d_{1}u_{0}x_{2}+\theta x_{1}^{2},\\ {\dot{x}}_{2} & = & d_{2}u_{1}-x_{2}, \end{array}$$

where θ is an unknown parameter. The original goal of this simulation is to design control inputs $u_{0}$, $u_{1}$ such that $(\eta \left(t\right),x_{0}\left(t\right),x_{1}\left(t\right),x_{2}\left(t\right))\to 0$ when t→∞. There is no loss of generality in assuming initial state $x_{0}\left(0\right)$ nonzero. Then system (47) is further divided into two subsystems:

(48) $$\begin{array}{ccc} \dot{\eta } & = & -\frac{\eta }{1+\eta^{2}}+x_{0}^{2},\\ {\dot{x}}_{0} & = & d_{0}u_{0}+\frac{1}{2}x_{0}+\frac{1}{2}x_{0}\frac{\eta }{1+\eta^{2}}, \end{array}$$

and

(49) $$\begin{array}{ccc} {\dot{x}}_{1} & = & d_{1}u_{0}x_{2}+\theta x_{1}^{2},\\ {\dot{x}}_{2} & = & d_{2}u_{1}-x_{2}, \end{array}$$

For system (48), it can be drawn a conclusion that the inverse dynamics $\dot{\eta }=-\frac{\eta }{1+\eta^{2}}+x_{0}^{2}$ is iISS, not ISS. In fact, choosing $V\left(\eta \right)=\ln\left(1+\eta^{2}\right)$, it is easy to testify that $\dot{V}\left(\eta \right)\leq -\beta \left(|\eta |\right)+\gamma (|x_{0}\left|\right)$, with $\beta \left(|\eta |\right)=\frac{2\eta^{2}}{{\left(1+\eta^{2}\right)}^{2}}$, $\gamma (|x_{0}\left|\right)=x_{0}^{2}$. Obviously, β is merely a positive definite continuous function, not belongs to $K_{\infty }$-function. Then according to theorem 1, the control law is constructed as $u_{0}=-cd_{0}\kappa \varphi_{0}\left(x_{0}\right)x_{0},\dot{\kappa }=\Gamma \varphi_{0}\left(x_{0}\right)x_{0}^{2}$. The parameters are picked up as c=0.1, $d_{0}=1$, $\varphi_{0}=10$, Γ=0.1. For system (49), it can be converted into ${\dot{z}}_{1}=z_{2}-\frac{{\dot{u}}_{0}}{u_{0}}z_{1}+\theta u_{0}z_{1}^{2}$, ${\dot{z}}_{2}=u_{1}-z_{2}$ by coordinate transformation (7) and (22). A reduced-order observer is proposed by ${\dot{\hat{z}}}_{2}=u_{1}-{\hat{z}}_{2}$. Define the error $e=z_{2}-{\hat{z}}_{2}$ and it satisfies $\dot{e}=-e$. Apparently, assumptions all hold. Then control inputs $u_{1}$ and adaptive law $\dot{\hat{\theta }}$ are obtained by the similar procedure of Section 3.2.

**Figure 1:**
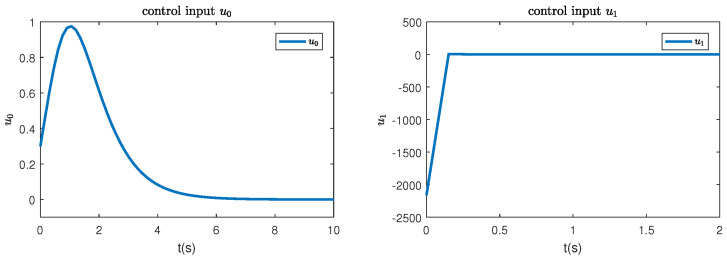
The control inputs $u_{0}$ and $u_{1}$.

**Figure 2:**
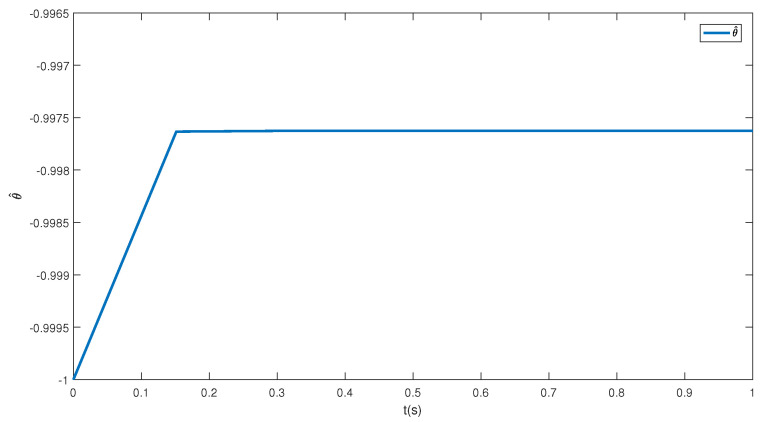
The unknown parameter estimation.

**Figure 3:**
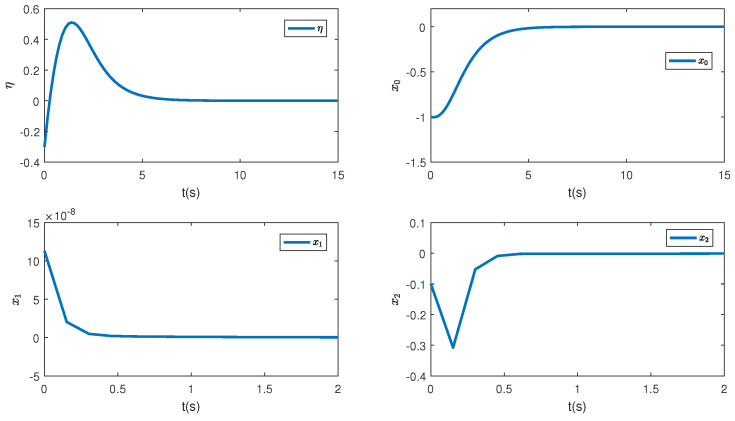
The corresponding states of the systems.

In this paper, a simulation is carried out based on the Matlab software of a 64-bit operation computer. Some values are set as $k_{1}=2$, $k_{2}=2$, $d_{1}=2$, $d_{2}=0.5$ and the initial condition is $\left(\eta \left(0\right),x_{0}\left(0\right),z_{1}\left(0\right),{\hat{z}}_{2}\left(0\right),\hat{\theta }\left(0\right),e\left(0\right),\kappa \left(0\right)\right)=(-0.3,-1,0.4,-0.3,-1,0.1,$ 0.3). Figure 1 depicts the control inputs designed by Theorem 1 and the reduced-order state observer-based backstepping method. The adaptive estimation of the parameter θ is given in Figure 2. It can be observed that the parameter estimation keeps stable after 0.15 s and nearly converges to its actual value. Figure 3 shows the inverse dynamic and states. As studied, η and $x_{0}$ are regulated to their original equilibriums after 5 s under the controller $u_{0}$. The states $x_{1}$ and $x_{2}$ are stable after 0.5 s under the controller $u_{1}$. This phenomenon is consistent with the tendency of controllers to some extent and the effectiveness of the proposed strategies is also shown.

**Figure 4:**
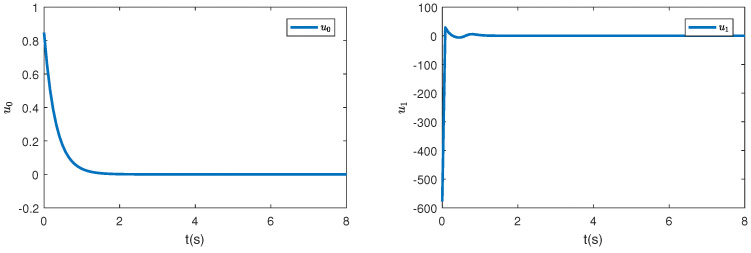
The control inputs $u_{0}$ and $u_{1}$ without inverse dynamics.

**Figure 5:**
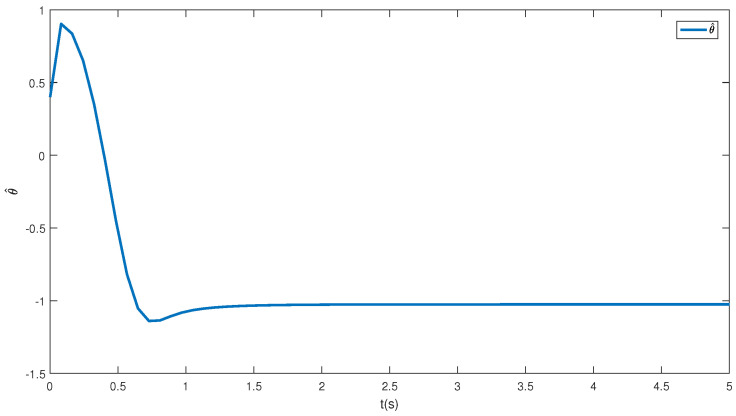
The unknown parameter estimation without inverse dynamics.

**Figure 6:**
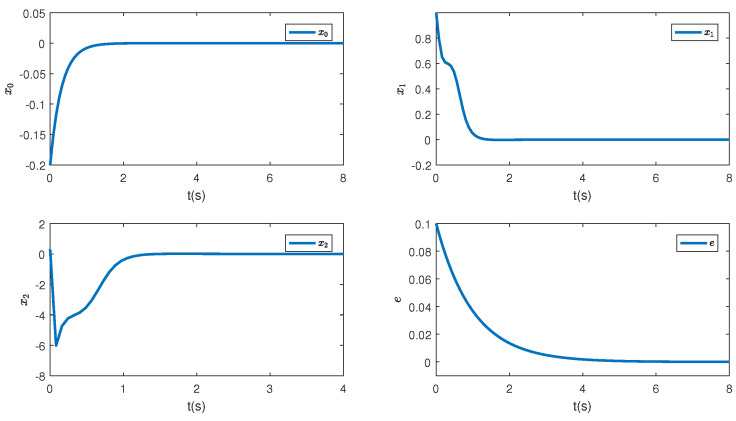
The system states without inverse dynamics.

To comprehensively illustrate the influence of the iISS inverse dynamics, a simulation is performed in a system without inverse dynamics. Figure 4, Figure 5 and Figure 6 are controllers, parameter estimation, states, and errors between $z_{2}$ and desired values, respectively. It can be noticed from Figure 6 that all the states converge to their equilibriums at similar times. Thus, it can be seen that the inverse dynamic affects the convergence time of the system.

## 6. Conclusions

In this paper, an adaptive output controller is designed for a class of nonholonomic chained systems with iISS inverse dynamics. Specifically, the nonholonomic system subjected to unknown virtual control directions and parameter uncertainty is divided into two subsystems. Two different controllers are designed for avoiding state finite escape and adaptive control objectives. One controller is designed by a switch strategy. Another is designed by combining a reduced-order state observer and backstepping method. However, there are still some problems unsolved in our work. Our future research will focus on more general nonholonomic systems with modeling uncertainties and environmental disturbances. And another coming issue is to take nonholonomic systems with iISS inverse dynamics and time delay into account.

## Data Availability

The data presented in this study are available on request from the corresponding author after obtaining permission of an authorized person.
